# Regulation of matrix metalloproteinases (MMPs) expression and secretion in MDA-MB-231 breast cancer cells by LIM and SH3 protein 1 (LASP1)

**DOI:** 10.18632/oncotarget.11720

**Published:** 2016-08-31

**Authors:** Marcel Endres, Susanne Kneitz, Martin F. Orth, Ruwan K. Perera, Alma Zernecke, Elke Butt

**Affiliations:** ^1^ Institute of Experimental Biomedicine II, University Medical Clinic of Wuerzburg, Wuerzburg, Germany; ^2^ Physiological Chemistry, Biozentrum, University of Wuerzburg, Am Hubland, Wuerzburg, Germany

**Keywords:** LASP1, matrix metalloproteinases, c-Fos, AP-1, extracellular matrix

## Abstract

The process of tumor invasion requires degradation of extracellular matrix by proteolytic enzymes. Cancer cells form protrusive invadopodia, which produce and release matrix metalloproteinases (MMPs) to degrade the basement membrane thereby enabling metastasis. We investigated the effect of LASP1, a newly identified protein in invadopodia, on expression, secretion and activation of MMPs in invasive breast tumor cell lines.

By analyzing microarray data of in-house generated control and LASP1-depleted MDA-MB-231 breast cancer cells, we observed downregulation of MMP1, -3 and -9 upon LASP1 depletion. This was confirmed by Western blot analysis. Conversely, rescue experiments restored in part MMP expression and secretion. The regulatory effect of LASP1 on MMP expression was also observed in BT-20 breast cancer cells as well as in prostate and bladder cancer cell lines.

In line with bioinformatic FunRich analysis of our data, which mapped a high regulation of transcription factors by LASP1, public microarray data analysis detected a correlation between high LASP1 expression and enhanced c-Fos levels, a protein that is part of the transcription factor AP-1 and known to regulate MMP expression. Compatibly, in luciferase reporter assays, AP-1 showed a decreased transcriptional activity after LASP1 knockdown.

Zymography assays and Western blot analysis revealed an additional promotion of MMP secretion into the extracellular matrix by LASP1, thus, most likely, altering the microenvironment during cancer progression.

The newly identified role of LASP1 in regulating matrix degradation by affecting MMP transcription and secretion elucidated the migratory potential of LASP1 overexpressing aggressive tumor cells in earlier studies.

## INTRODUCTION

The dissemination of cancer cells from a primary tumor to a distant site, known as metastasis, is a very complex process. An essential step requires the degradation of the extracellular matrix (ECM) allowing tumor cells to invade surrounding tissue and to disseminate in blood and lymph vessels [1]. This process is primarily influenced by proteinases such as matrix metalloproteinases (MMPs), secreted by invadopodia of the tumor cell, respectively by podosomes in monocytic cells [2]. MMPs are grouped by their historical substrate specificity (mainly collagen, gelatin and stromelysin) and their cellular localization. All MMPs are synthesized and secreted as zymogens. Hence, they require extracellular activation [3]. Earlier studies investigated the expression of MMPs in primary human breast cancer and breast cancer cell lines, concluding an enhanced expression and secretion of MMP1, -2, -3 (only in cell lines), -7 (with restriction), -10, -11, -12, -13, -14, -23, -27 and -28 in highly metastatic cell lines [4, 5]. MMP8, -10, -12 and -27 are related to higher tumor grade in breast cancer. A very recent meta-analysis identified MMP2 and -9 overexpression to be associated with higher risk of poor prognosis [6].

Both, podosomes and invadopodia are composed of an F-actin-rich core that is surrounded by adhesion and scaffolding proteins like cortactin, cofilin, dynamin, Arp2/3, tyrosine kinase substrates and the MMPs, mainly MMP2, MMP9 and the membrane form MT1-MMP (also known as MMP14) [7]. In 2012, Stölting *et al*. identified a new scaffolding protein in podosomes of macrophages – LIM and SH3 protein 1 (LASP1) [8]. LASP1 is an ubiquitously expressed, actin-binding scaffolding protein, known to regulate cytoskeleton architecture and cell migration [9, 10]. Various cytoskeleton proteins have been identified that bind to LASP1: F-actin [11], kelch-related protein [12], zyxin [13], lipoma preferred partner [11], zona occludens protein 2 (ZO2) [14], dynamin [14], VASP [11], CRKL [15] and CXCR2 [16], respectively.

In macrophages, LASP1 was found in the ring structure of podosomes [8]. Knockdown of LASP1 affected size, number and lifetime of the podosomes and, moreover, inhibited matrix degradation capacity. However, the molecular function of LASP1 was not dissected in more detail.

Next to its physiological role as a structure protein, LASP1 has been implicated in cell migration and invasion. In hepatocellular carcinoma (HCC), a functional repression effect of p53 on LASP1 via a p53 response element is observed [17] while increased LASP1 expression is seen after urokinase-type plasminogen activator (uPA) up-regulation [18]. Overexpression of the protein is detected in various tumor entities concomitant with a reduced overall survival of the patients in prostate cancer [19], HCC [20], colon cancer [21], gastric cancer [22] and medulloblastoma [23]. Knockdown of LASP1 leads to reduced proliferation and migration of these tumor cells. Furthermore, a nuclear LASP1 positivity is observed in several cancer entities and is correlated with worse patient outcome, at least, in breast cancer, and a transcriptional role for the protein is discussed [14, 24].

In the present study, we investigated the effect of LASP1 on expression, secretion and activation of MMPs in MDA-MB-231 breast cancer cells since LASP1 overexpression could promote MMP activity in podosomes and invadopodia and hence, by analogy to its observed role in podosomes of macrophages, modulate matrix degradation and invasive potential of aggressive tumors.

## RESULTS

### LASP1 is involved in matrix degradation by invadopodia in breast cancer cells

**Figure 1:**
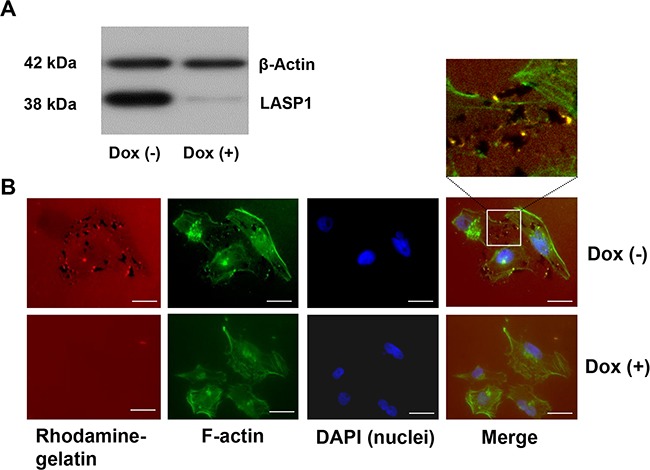
Knockdown of LASP1 decreases matrix degradation capacity of invadopodia in human breast cancer cells **A.** Western Blot analysis of LASP1 and β-actin (loading control) of MDA-MB-231-shLASP1 cells before and after doxycycline treatment. **B.** Representative omnifocal microscopy of stably transfected MDA-MB-231shLASP1 cells before (Dox(−)) and after LASP1 knockdown (Dox(+)), seeded on rhodamine-labeled gelatin, fixed after 20h and stained for F-actin with Oregon Green 448 phalloidin and DAPI. Matrix degradation is visible as dark spots by concomitant loss of the fluorescent label. For merged Dox (−) images, a high magnification with yellow spotted invadopodia is shown. Scale bars represent 20 μm.

In analogy to our earlier experiments with macrophages [8], we analyzed LASP1 influence on matrix degradation by invadopodia in breast cancer cells. Therefore, we generated MDA-MB-231-shLASP1 cells, a highly LASP1 expressing invasive breast cancer cell line stably transfected with doxycycline-inducible shRNA against LASP1. The shRNA recognizes a sequence in the 5′non-coding region of LASP1. Knockdown was confirmed by Western blot analysis and showed an average silencing of LASP1 of about 80-90% after 72 hours (Figure 1A).

For the assay, control cells and LASP1-depleted MDA-MB-231-shLASP1 cells were seeded on coverslips coated with rhodamine-labeled gelatin. After 20 hours, cells were fixed and stained with phalloidin green (F-actin) to visualize the cell outline and the invadopodia, and with DAPI for nucleus staining. 77-80% of the control cells showed distinct gelatin degradation, visible as black dots in the red matrix while only 15-20% of the LASP1 knockdown cells exhibited matrix degradation. Representative pictures are shown in Figure 1B. In the higher magnification of the merged Dox (−) image, invadopodia are visible as yellow spots just next to the degraded matrix. It is interesting to note that LASP1 depleted cells with a low cellular LASP1 concentration, exhibit a more profound physiological cytoskeletal network with defined actin stress fibres than the based tumor cell with accumulated intermediate filaments [25].

### LASP1 regulates expression of MMP1, -3 and 9 in breast cancer cells

In a second step we analyzed whether loss of LASP1 is affecting gene expression of proteins involved in invadopodia activity and performed differential gene expression analysis of breast cancer MDA-MB-231-shLASP1 control and knockdown cells. Changes in gene expression comprising selected matrix metalloproteases upon LASP1 knockdown in MDA-MB-231 cells are summarized in Table 1. A list of 39 key genes regulated by LASP1 is found in Supplementary Table S1. We observed a decent downregulation of MMP1 and -3.

**Table 1:** Changes in matrix metalloproteinase gene expression upon LASP1 knockdown in MDA-MB-231 cells

Gene symbol	Fold change	p-Value	Accession number
LASP1	−5.40	0.00001	NM_006148
MMP1	−1.68	0,0015	NM_002421
MMP3	−1.77	0.018	NM_002422
MMP9*	−1.90	0.001	NM_004994
MMP2	1.16	0.079	NM_004530
MMP7	−1.03	0.139	NM_002423
MMP12	1.14	0.197	NM_002426
MMP13	−1.03	0.224	NM_002426
MMP14	1.01	0.538	NM_004995
MMP19	1.02	0.085	NM_004994
TIMP2	−1.02	0.722	NM_003255

Fold change (FC) = LASP1-knockdown cells/control cells (if FC < 1, the reciprocal FC is shown with a minus sign).

* Ref.: 26.

At that time, online microarray data by Duvall-Noelle showed downregulation of MMP9 after LASP1 knockdown in breast cancer cells [26]. Therefore, this metalloprotease was included in our further experiments. For completion, we added MMP2 as a second gelatinase and MMP14 (MT1-MMP) as a prototypical membrane-associated proteinase to our study [27].

**Figure 2:**
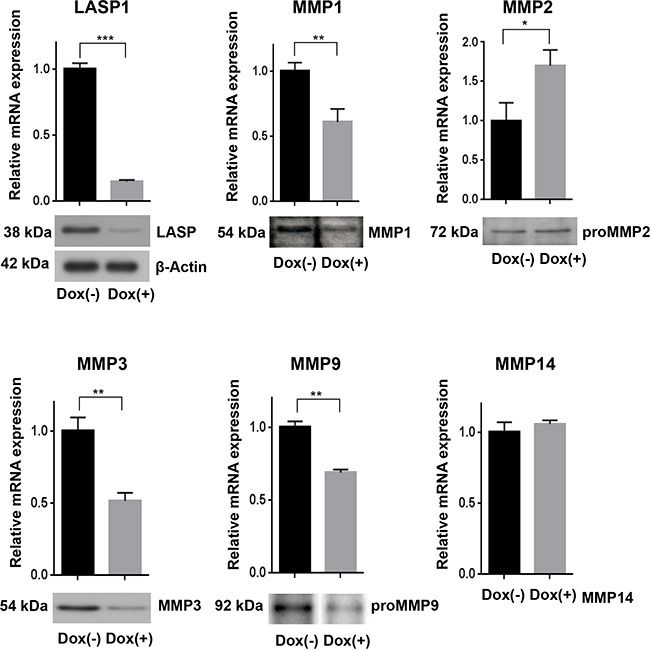
Loss of LASP1 in breast cancer cells affects gene expression of matrix metalloproteinases qRT-PCR in human MDA-MB-231-shLASP1 cells was performed with SYBR Select Master Mix. Bar graphs show the relative mRNA expression, calculated by the comparative ddCT-method and normalized to the housekeeping gene ribosomalprotein large P0 (RPLP0). LASP1 depletion decreased MMP1, MMP3, and MMP9 gene expression while MMP2 gene expression increased. MMP14 mRNA was not affected by LASP1 knockdown. *p<0.05; **p< 0.01; ***p<0.001; n=5. Western Blot analysis confirmed LASP1 knockdown concomitant with impaired MMP1, -3, and -9 protein levels while MMP2 protein expression was not affected. The LASP1 knockdown and β-actin loading control shown applies to all other blots as well. Representative blots of five independent experiments are shown.

To validate the microarray data, we performed qRT-PCR assays and Western blots for assessing mRNA levels and protein concentration of the matrix metalloproteases before and after LASP1 knockdown in the breast cancer cell line. The reduced gene expression of MMP1, -3 and -9, seen after LASP1 knockdown by microarray analysis, could be confirmed by qRT-PCR and was also detected on protein basis by Western blots with MMP-specific antibodies (Figure 2). For MMP2, we measured a faint but significant increase in mRNA level, however, this observation could not be confirmed by immunoblots (Figure 2). MMP14 mRNA was not affected by LASP1 (Figure 2). Effects on recruiting MMP14 to podosome membranes by LASP1 had already been excluded earlier [8].

To rule out any off-target effects induced by the lentiviral infection, we repeated the knockdown experiments using specific siRNA against LASP1 covering the bp 80-100. Transient LASP1 knockdown in MDA-MB-231 cells resulted in a similar MMP1, -3, and -9 downregulation as observed for stable shRNA-LASP1 transduction (Supplementary Figure S1).

### LASP1 regulates secretion of MMPs into the extracellular matrix

Next to the regulation of MMPs on the transcriptional level by LASP1, the scaffolding function of the protein allows regulation of secretory processes. We therefore analyzed MMP secretion and measured protease concentration in the cell and in the conditioned medium before and after LASP1 knockdown in MDA-MB-231-shLASP1 cells.

**Figure 3:**
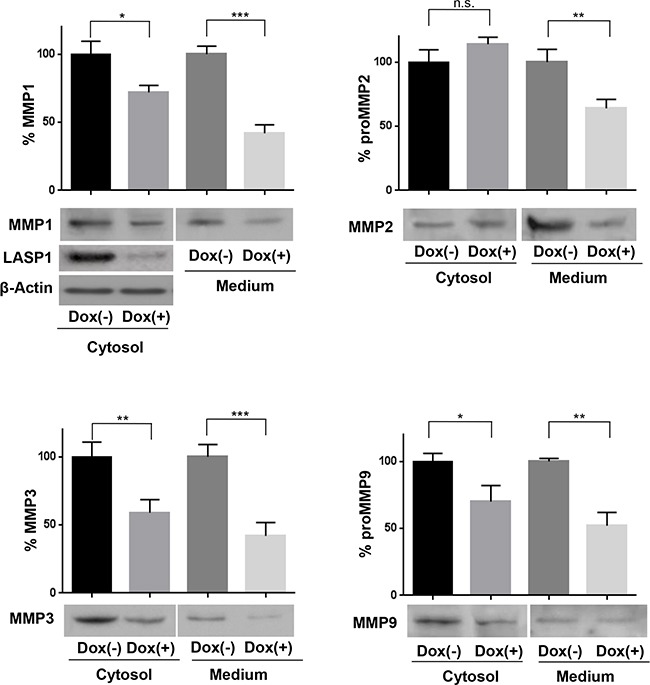
Loss of LASP1 impairs matrix metalloproteinases secretion by breast cancer cells Cell lysate and adapted corresponding serum-free conditioned medium were harvested from MDA-MB-231-shLASP1 untreated control cells (Dox (−)) and LASP1 knockdown cells (Dox (+)) and subjected to Western blot analysis using MMP-specific antibodies. Representative blots are shown. The LASP1 knockdown and β-actin control shown for MMP1 expression applies to all other blots as well. Bar graphs summarize the results of five independent experiments (*p<0.05; **p<0.01; ***p<0.001; n.s. not significant). Compared to control cells, knockdown cells showed relative less abundant protein levels in the conditional medium and the cell lysate.

While depletion of LASP1 resulted in an intracellular suppression of MMP1, -3, and 9 by 33% (± 15), a much more pronounced reduction (48 ± 16%; p=0.03, n=15) is seen when evaluating the secreted pools (Figure 3). This difference is most obvious for MMP2. While intracellular expression of MMP2 is not affected by LASP1 knockdown, protease secretion is reduced by 36 % (Figure 3), indicating a role of LASP1 in matrix metalloprotease release, in addition to transcriptional regulation.

### Rescue experiments confirm the role of LASP1 on MMP expression and secretion

**Figure 4:**
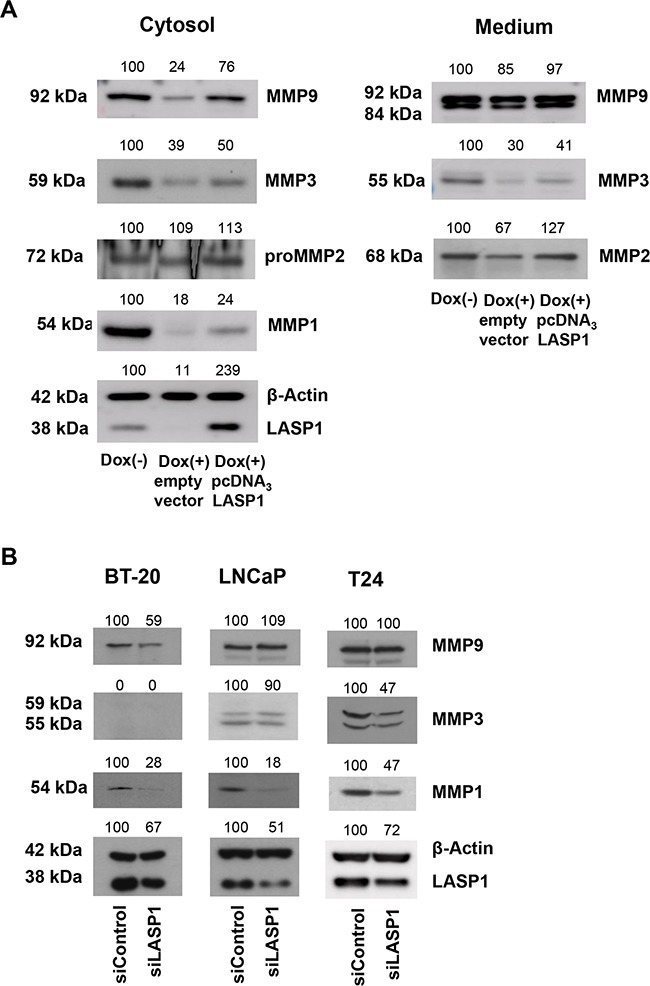
LASP1 rescue experiments and MMP expression in other tumor entities **A.** For LASP1 rescue experiments, MDA-MB-231-shLASP1 cells were treated for 4 days with doxycycline. After 2 days, transfection with pcDNA_3_-LASP1 restored LASP1 expression. Cell lysate and adapted corresponding serum-free conditioned medium were harvested from MDA-MB-231-shLASP1 untreated control cells (Dox (−)), LASP1 knockdown cells (Dox (+)) and LASP1 restored cells (Dox (+)/pcDNA_3_) and were subjected to Western blot analysis. Representative blots of two independent experiments are shown, confirming successful knockdown and rescue of LASP1, concomitant with reduced and restored MMP expression and secretion. **B.** LASP1 knockdown in BT-20 breast cancer, LNCaP prostate and T24 bladder cancer cell lines. LASP1 specific siRNA transfection leads to impaired MMP1, -3, and -9 expression in the cell lysate. Numbers above the blots indicate percentage of protein expression after LASP1 knockdown, respectively reconstitution, compared to 100% control. Representative Western blots of three independent experiments are shown. β-actin served as loading control.

To confirm the pivotal role of LASP1 on MMP levels, we performed LASP1 rescue experiments by transfecting LASP1 depleted MDA-MB-231-shLASP1 cells with pcDNA_3_-LASP1. Since the shLASP1 is designed against the 5′non-coding region of LASP1, the coding LASP1 mRNA of the pcDNA_3_ construct is not affected. Western blots demonstrate LASP1 knockdown after doxycycline treatment and successful reconstitution/overexpression of the protein (Figure 4A Cytosol). Concurrently, MMP1, -3 and -9 protein levels decreased after LASP1 silencing while restoring of LASP1 recovered MMP concentrations in the cell and in the medium, at least in part (Figure 4A).

Again, cellular MMP2 concentration was not affected by LASP1 depletion or by protein recovery, while in the conditioned medium LASP1 knockdown led to reduced MMP2 levels that are restored after LASP1 overexpression (Figure 4A Medium) supporting the active role of LASP1 in MMP secretion process.

### Effects of LASP1 on MMPs is not restricted to MDA-MB-231 cells

Since LASP1 overexpression and worse patient outcome has been described in several cancer entities, we next checked whether LASP1 knockdown is also affecting MMP expression in the primary, non-invasive, BT-20 breast cancer cell line as well as in T24 bladder and LNCaP prostate cancer cell lines, known to express high levels of LASP1 [19, 28]. Knockdown of LASP1 by specific siRNA transfection resulted in decreased, however not consistent, protein levels of MMP1, -3 and -9 in the cell lysate (Figure 4B). While MMP3 is not expressed in BT-20 cells [5], MMP9 is not affected by LASP1-depletion in LNCaP and T24 cells. On the other hand, moderate knockdown of LASP1 by 50% in the prostate cell line reduces MMP1 concentration down to 18%. Overall, LASP1 effects on MMP expression are not restricted to breast cancer cells but are also not regulated alike in all cells.

### LASP1 knockdown does not impair MMP activity

To assure temporal and spatial control of MMP activity, MMPs are synthesized as inactive pro-enzymes (pro-MMPs). The activity can be analyzed by zymography, an electrophoretic technique for the detection of hydrolytic enzymes, based on the substrate repertoire of the enzyme [29].

**Figure 5:**
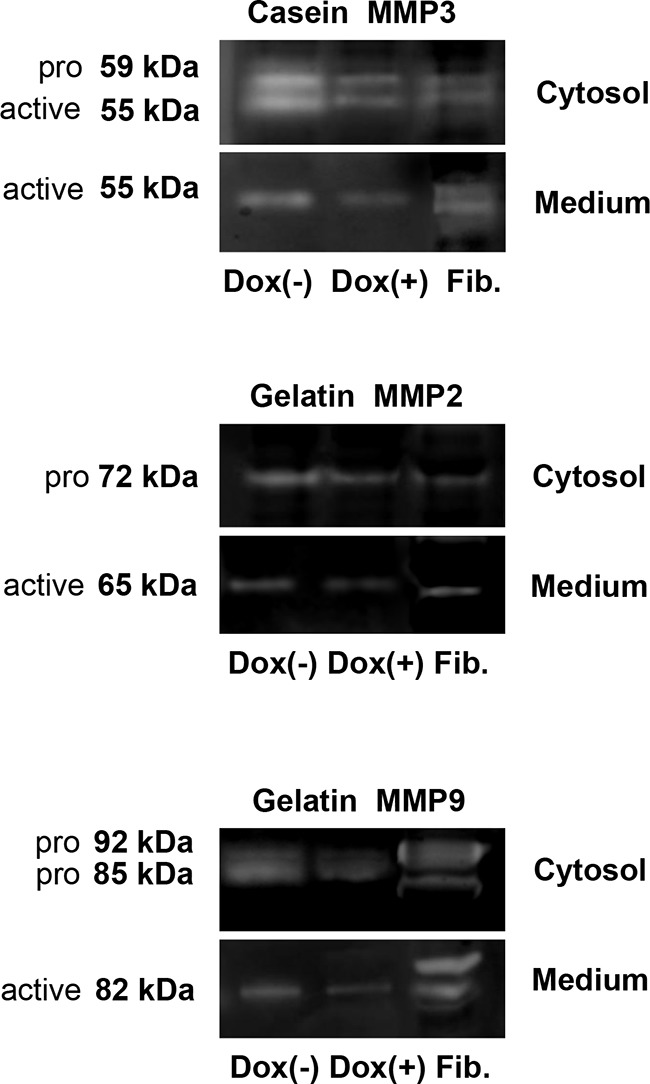
Gel zymography of cell lysate and conditional medium from MDA-MB-231-shLASP1 cells Samples of cell lysate (5×10^4^ cells) and conditional media from 1×10^6^ MDA-MB-231-shLASP1 control (Dox (−)) and LASP1 knockdown (Dox (+)) cells were used for casein- (MMP3) and gelatin-zymography (MMP2 and -9). Enzymatic activity was detected in all samples. Human uterine fibroblasts (Fib.) served as positive control. Only the active forms are detected in the medium. Data are representative for three independent experiments.

We collected cells and conditioned media from untreated and doxycycline-treated MDA-MB-231-shLASP1 cells and performed zymography. MMP3 is best detected on casein gels. As seen in Figure 5, upper panel, the pro- and active form of MMP3 in the cell lysate can be distinguished by its molecular weight at 59 and 55 kDa, respectively. Only the active form is secreted.

Gel zymography with gelatin as a substrate can identify the latent (proMMP2: 72 kDa; proMMP9: 92 kDa) and active (MMP2: 65 kDa; MMP9: 82 kDa) gelatinases (Figure 5, middle and lower panel, respectively). A reported 85 kDa pro-form of MMP9 in breast tumor cells, lacking complex carbohydrates [30], is also visible in our zymograms while control fibroblasts solely exhibit the 92 kDa pro-form band. Only the active forms are detected in the conditioned medium.

Both substrates allow the detection of MMP1 at 54 kDa (pro-form) and 44/41 kDa (active form), however, the signal will be very weak because gelatin and casein are not preferred substrates. We failed to see any enzymatic activity for MMP1 around 40 kDa. The pro-form overlapped with MMP3.

As already observed by Western blot with MMP-specific antibodies, the overall amount of MMP2, -3, and -9 is clearly reduced in the medium of doxycycline-treated cells and a decreased level of MMP3 and -9 is also detected in the cell lysate of LASP1 knockdown cells compared with untreated cells. Overall, LASP1 knockdown is not affecting MMP activity in MDA-MB-231 cells.

### Knockdown of LASP1 suppresses AP-1 transcriptional activity

MMP gene expression is primarily regulated at the transcriptional level. We therefore analyzed the transcriptional regulation of the top 39 regulated genes after LASP1 knockdown in our microarray data set by functional enrichment analysis using FunRich software (*Functional Enrichment analysis tool* (http://www.funrich.org)) [31]. Data revealed a more than 2-fold enrichment of genes with c-Jun and c-Fos transcriptional activity, among them MMP1.

Transcription factor database research identified AP-1 binding site being the common promoter site present in *MMP1*, *-3*, and -*9* but not in *MMP2* and *-14* (http://www.sabiosciences.com/chipqpcrsearch.php).

**Figure 6:**
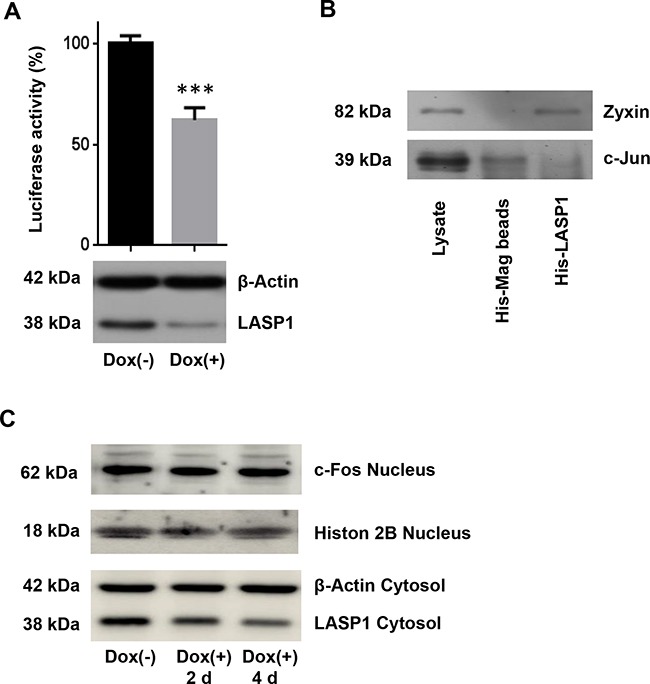
Luciferase reporter assay for AP-1 transcriptional activity and His-LASP1 pulldown **A.** MDA-MB-231-shLASP1 cells, pre-treated 3 days with or without doxycycline, were infected with AP-1 binding site reporter lentiviruses to detect endogenous AP-1 activity, and with Renilla-luciferase plasmids for internal standard. Equal numbers of cells were then analyzed for both, firefly and Renilla luciferase activity. Data presented show firefly luciferase activity after normalization with Renilla luciferase and further normalized to control; *** p<0.001 (n =3). Data show reduced AP-1 activity after LASP1 knockdown. **B.** Western blot analysis of c-Jun after His-tagged LASP1 pulldown in MDA-MB-231-shLASP1 cell lysate. Specific binding of zyxin to LASP1 served as positive control. No specific binding of c-Jun to LASP1 is observed. **C.** Western blot analysis of c-Fos expression in MDA-MB-231-shLASP1 nuclear extract after 2 and 4 days of doxycycline treatment. LASP1 knockdown is not affecting c-Fos protein concentration. A representative blot of three independent experiments is shown. Histon 2B served as nuclear loading control. Western blot analysis of the cytosolic fraction revealed time-dependent LASP1 knockdown. β-actin served as cytosolic loading control.

AP-1 is a heterodimer that comprises members of the proto-oncogene c-Jun and c-Fos protein family and may form ternary complexes with transcriptional co-factors [32]. We therefore tested transcriptional activity of AP-1 in control and LASP1 knocked-down MDA-MB-231-shLASP1 cells by using a luciferase reporter assay with a mixture of inducible AP-1 responsive firefly luciferase construct and constitutively expressing Renilla luciferase construct as internal standard. Cells depleted of LASP1 showed a 40% decreased AP-1 transcriptional activity compared with LASP1 expressing control cells (Figure 6A).

Since earlier co-immunoprecipitation experiments clearly demonstrated binding between c-Jun and LIM-domain proteins to activate AP-1 [33] we performed immunoprecipitation experiments with LASP1 and c-Jun specific antibodies (data not shown) as well as pulldown assays with GST-tagged- and His-tagged-LASP1 in MDA-MB-231-shLASP1 cell lysate and with purified nucleus preparation. Specific binding of zyxin to LASP1 served as positive control (Figure 6B). However, all efforts to demonstrate a direct interaction between LASP1 and c-Jun failed (Figure 6B); only unspecific binding of c-Jun to sepharose A/G beads was observed, suggesting no direct effect of LASP1 on AP-1 transcriptional activity.

While analysis of microarray data for primary breast cancers revealed significant lower c-Fos mRNA levels in tumor samples with low LASP1 expression (p<0.001, Supplementary Table S2), the analysis of our microarray data set pointed to up-regulation of *c-Fos* transcription by LASP1 depletion (Supplementary Table S1). However, Western blot analysis of MDA-MB-231-shLASP1 nuclear extract −/+ doxycycline treatment after 2 and 4 days could not verify regulatory effects of LASP1 on c-Fos protein level (Figure 6C), suggesting a more complex regulatory function of LASP1 on MMP expression.

## DISCUSSION

Metastatic dissemination of cancer cells by degrading the extracellular matrix of basement membranes, tumor stroma, and blood vessels is the leading cause of mortality in patients with malignant cancers. This process is facilitated by the formation of invadopodia, ventral membrane protrusions formed by tumor cells that produce and release matrix metalloproteinases to perforate the native basement [34].

LASP1, the newly identified regulatory protein in invadopodia in this study, is overexpressed in numerous tumor entities and correlates with tumor aggressiveness [9]. The present data provide an important clue on the pathophysiological role of LASP1 on MMP regulation and hence metastasis in aggressive cancer cells.

MMPs are regulated on several levels: expression, trafficking, zymogen activation, and enzyme inhibition/deactivation.

By gel zymography, we excluded any effect of LASP1 on MMP enzymatic activity. qRT-PCR and Western blot analysis revealed reduced levels of MMPs on mRNA and protein levels upon LASP1 knockdown and effects on secretion. Impaired release was most obvious for MMP2 (Figure 3), a proteinase with known functions in breast cancer invasion and metastasis [35]. Major MMP2 activation occurs extracellularly through the formation of a proMMP2-MMP14-TIMP2 complex. While LASP1 is not regulating MMP2 transcription (see microarray results in Table 1) or activation (neither MMP14 nor TIMP2 mRNA levels are altered by LASP1 knockdown; see Table 1), MMP2 secretion is strongly reduced in LASP1 depleted breast cancer cells. This negative impact of LASP1 knockdown on protease release is also observed for MMP1, - 3 and -9, although to a minor extend (Figure 3). Release of soluble MMPs involves packaging of the enzymes in small vesicles that are then directed towards invadopodia along microtubules by the molecular motor protein kinesin and secreted in vesicles into the surrounding tissue [36, 37]. This process is similar to the release of melanin vesicles in melanoma cells, a mechanism in part regulated by LASP1 [38]. In these cells, depletion of LASP1 resulted in a reduction of melanosome vesicle shedding - most likely by loss of the LASP1-dynamin bridging complex during vesicle budding [38].

Latest studies revealed a fractionated release of vesicular content, called “kiss-and-run” process. This mechanism is shown for neurotransmitter release but is also discussed for other exocytotic responses [39]. In this process, dynamin, in cooperation with other proteins, promotes opening of the pore during vesicle content release.

Both mechanisms are in agreement with a regulatory function of LASP1 in MMP vesicle secretion in breast cancer as earlier immunoprecipitation studies demonstrated a distinct LASP1-dynamin [14] and LASP1-actin [40] interaction in breast cancer cells and a co-localisation of LASP1, F-actin and dynamin in podosomes [8, 41].

On the transcriptional basis, we observed reduced mRNA expression of MMP1, -3 and -9 after LASP1 knockdown in MDA-MB-231 breast cancer cells that could be confirmed on protein level. Transcriptional up-regulation of MMPs involves activation of several well-known factors including p53, CREB1, PEA3, AP-1, SP1 and C/EBP [42]. Thereof, AP-1, a stable heterodimer composed of c-Jun and c-Fos protein and thereby enhancing DNA binding activity [43], was the most promising candidate to regulate MMPs by LASP1 as the AP-1 binding site is the only common proximal promoter site in MMP1, -3 and -9 and present 2-3 times close to a typical TATA box, while this site is absent in the LASP1 non-regulated matrix metalloproteases MMP2, -14 and -19 or only present singularly in MMP7, -11, and -13.

Furthermore, analysis of publically available microarray data of low and high LASP1-expressing breast cancer probes revealed a significant higher c-Fos expression in LASP1-high tumor samples (p<0.001) (Supplementary Table S2).

In agreement with these data, our results showed that depletion of LASP1 in breast cancer cells reduced AP-1 transcriptional activity. However, despite several reports of LIM-domain containing proteins as co-activators of transcription factors (LPP is a co-activator for the transcription factor PEA3 [44], LimD1 regulates AP-1 activation through an interaction with Traf6 [45], and the LIM domain protein nTRIP6 interacts with AP-1 and enhances its transcriptional activity [46]) we could not proof any direct interaction of LASP1 with c-Jun (Figure 6B) and there was no effect of LASP1 on c-Fos expression (Figure 6C) as primarily assumed when analyzing the microarray data set of MDA-MB-231-shLASP1 (Supplementary Table S1). So far, we have no mechanistic explanation of how LASP1 is regulating AP-1 and MMP expression.

With respect to nuclear LASP1 effects on AP-1, it is interesting to note, that shuttling of LASP1 into the nucleus depends on LASP1 binding to zona occludens protein 2 (ZO-2) [14]. ZO-2 is a multidomain protein that regulates the assembly of tight junctions and has also been reported to transiently accumulate in the nucleus and suppresses AP-1 gene expression by binding to the transcription factor protein complex c-Jun/c-Fos, mainly with its proline rich domain. [47, 48]. Since c-Jun/c-Fos and LASP1, both bind to the same C-terminal region in ZO-2, it is tempting to speculate that overexpression (as observed in aggressive tumors) and binding of LASP1 to ZO-2 prevents inhibitory association of c-Jun/c-Fos with ZO-2, and hence, free AP-1 transcription factor complex activates MMP gene expression.

In completion of this aspect, it should be noticed that LPP, the co-activator of the transcription factor PEA3 [44] is a binding partner of LASP1, and a definite LASP1/LPP interaction has been detected in the nucleus of MDA-MB-231 cells [14]. However, a PEA3 promoter binding site is also present in MMP2, a matrix metalloprotease not regulated by LASP1, at least not in MDA-MB-231 cells.

Further experiments will address this interesting, potential regulatory impact of LASP1 on transcriptional activity and cancer progression.

Finally, we analysed expression of MMPs in human breast cancer and metastatic samples (as MDA-MB-231 is a triple-negative metastatic cell line) in publically available microarray data (Supplementary Table S2). For MMP9, a significant correlation between high LASP1 expression and enhanced MMP mRNA levels was observed (p=0.003) and could be confirmed in metastatic breast cancer (p=0.04). However, we could not verify our results for MMP1 and -3 in public data. But, as our expression data analyses revealed significantly lower expression of MMPs in metastases than in primary breast tumors, MMPs might be only temporary relevant for metastasis. Therefore the effect of LASP1 on MMPs might also be transient and, hence, not easily accessible with public expression data.

MMP regulation by LASP1 is not restricted to breast cancer cells. Knockdown of LASP1 in prostate and bladder cancer cell lines also resulted in decreased MMP levels although with different impact.

Since a correlation between LASP1 overexpression and worse patient outcome has not only been described in bladder [28, 49] and prostate cancer [19, 50] but also in ovarian cancer [51], medulloblastoma [23], colon cancer [21], esophageal squamous cell carcinoma [52], kidney cancer [53], and liver cancer [20], it is tempting to speculate that up-regulation of MMP expression and secretion by enhanced LASP1 concentrations is a major factor in tumor aggressivity.

Recently, two additional papers studied the role of nuclear LASP1 function and revealed co-localization of LASP1 with proteins of the epigenetic machinery; especially UHRF1, DNMT1, G9a and the transcription factor Snail1 [26]. Snail1 is known to amplify MMP gene expression of MMP1, -2, -7 and -14 in hepatocellular carcinoma cells [54]. The authors discuss a stabilization of Snail1 by LASP1 association.

Moreover, LASP1 is discussed to be involved in inducing TGF-β mediated epithelial-mesenchymal transition (EMT) by regulating S100A4 expression and promoting cell invasiveness [55]. EMT is an important process during development by which epithelial cells acquire mesenchymal, fibroblast-like properties and show reduced intercellular adhesion and increased motility. Physiopathological transition occurs during the progression of tumors, endowing cancer cells with increased motility and invasiveness. In this respect, LASP1 has recently been characterized as a new binding partner for vimentin, a marker protein for cells undergoing EMT [56].

A more detailed bioinformatic analysis of the microarray data set of MDA-MB-231-shLASP1 by functional enrichment analysis (FunRich analysis) revealed that 35 of the top 39 regulated genes can be mapped to transcription factors, mainly DLX5, FOX, HOX, ETS1 and c-JUN/c-FOS, hence supporting an effect of LASP1 on AP-1. Further analysis failed to elucidate the mechanism by which LASP1 regulates MMPs and invadopodia. Statistically significant altered proteins play a role in lipid metabolism, signal transduction and uPAR pathway. Functional analysis showed that mainly immune response and inflammatory pathways as well as cell-to-cell signaling events are overrepresented in this set: interferon alpha/beta signaling, glucocorticoid receptor network, IL2-mediated signaling events and regulation of E-/N-cadherin adherens junction stability and disassembly.

In conclusion, we identified LASP1 as a novel regulator protein of invadopodia in breast cancer cells being involved in extracellular matrix degradation by matrix metalloproteases. LASP1 enhances secretion of MMP favoring distant metastasis. Furthermore, LASP1 induces MMP gene expression by somehow indirect regulation of the AP-1 transcription factor. Further investigation is needed to determine the regulatory transcriptional mechanisms of LASP1.

## MATERIALS AND METHODS

### Cell culture

The human breast cancer cell lines MDA-MB-231 (triple-negative breast cancer metastasis) and BT-20 (primary tumor) as well as the prostate cancer cell line LNCaP and the bladder cancer cell line T24 were purchased from ATCC (Manassas, VA) and grown in RPMI 1640-GlutaMAX™ (Gibco BRL, Wiesbaden, Germany) supplemented with 10 % FBS and Pen/Strep (PAA, Coelbe, Germany). Cells were cultivated in a 5 % CO_2_ atmosphere at 37°C in a fully humidified incubator.

### Lentiviral transduction

MDA-MB-231 cells expressing inducible silencing LASP1-shRNA were generated using lentiviral infection. The pTRIPZ-shRNAmir plasmid (V2THS-64686, Open Biosystems, Huntsville, USA) expressing LASP1-specific shRNA (sequence 5′- GGCAAGTGGAATATCTTATAT-3′ in the non-coding 5′-region) was packaged into lentiviral particles in HEK297T cells and MDA-MB-231 cells were infected with the particles following the manufacturer's protocols. Stable cell lines were selected for resistance to puromycin (2 μg/ml) and single cells were selected to generate clonal cell lines with a LASP1 knock down ≥80 % after three days of doxycycline treatment (0.5 μg/ml).

### Rescue experiments

For rescue experiments, MDA-MB-231-shLASP1 cells were cultured for an overall time of 4 days with and w/o doxycycline. After 2 days, transfection was performed by Amaxa electroporation (program X013, Lonza, Cologne, Germany) using 1×10^6^ cells and 0.25 μg pcDNA_3_-LASP1 wt construct or empty vector [11]. After transfection, cells were seeded for another two days with and w/o doxycycline.

### siRNA transfection

Transfection for LASP1 knockdown was performed using metafectene (Biontex Laboratories GmbH, Martinsried, Germany) according to the manufacturer's protocol for suspension culture. Briefly, suspended cells were used at a density of 1×10^5^ cells/ml and incubated directly with the pre-incubated mixture of 6 μl metafectene/ml cell suspension and control siRNA (20 nM final, AllStar negative control, Qiagen, Hilden, Germany) or LASP1 siRNA (20 nM final, 5′-AAG CAT GCT TCC ATT GCG AGA -3′; bases 80-100). Cells were cultured at 37°C under 5 % CO_2_ atmosphere for 72 h. Knockdown was controlled by Western blot.

### Western blot

Protein lysates were subjected to 10% SDS-PAGE and blotted onto nitrocellulose membrane (GE Healthcare, Freiburg, Germany). Equal amount of cells (1×10^4^) were analyzed by immunoblotting with the following antibodies: self-generated LASP1 [57] (diluted 1:2000); anti-β-Actin (Santa Cruz #1616, CA, USA, diluted 1:3000); MMP1 (Abcam #38929, Cambridge, UK, diluted 1:500); MMP2 (Santa Cruz #10736, diluted 1:100); MMP3 (Abcam #52915, diluted 1:1000); MMP9 (Merck Millipore #19016, MA, USA, diluted 1:500); c-Fos (Santa Cruz #52, diluted 1:200); c-Jun (Abcam #31419, diluted 1:500); monoclonal zyxin (Synaptic Systems, Göttingen, Germany, diluted 1:200); Histon 2B (Santa Cruz #8650, diluted 1:200).

Before overnight incubation with primary antibodies, membranes were blocked with 5 % dry milk (Bio-Rad, Munich, Germany) in TBS-T for 1h at RT. Immunoblots were probed with a secondary horseradish peroxidase conjugated antibody purchased from Bio-Rad and developed by using the enhanced chemiluminescence reagent (GE Healthcare). Chemiluminescence images were taken using the Amersham Imager 600 (GE Healthcare) and quantified by the Image QuantTL software (GE Healtcare).

### RNA isolation and quantitative real-time PCR

RNA isolation was carried out with pegGold Total RNA Kit (Peqlab, Erlangen, Germany) according to the manufacturer's instructions. 0.5 μg of total RNA was reverse-transcribed with the High-Capacity cDNA Reverse Transcription Kit (Life Technologies, Darmstadt, Germany). Quantitative real time PCR (qRT-PCR) was performed on a ViiA7 with SYBR Select Master Mix (Life Technologies). Relative mRNA expression was calculated by the comparative ΔΔCt-Method normalized to the housekeeping gene ribosomal protein, large, P0 (RPLP0). Primer sequences are listed in Supplementary Table S3. All primers had melting temperatures of 58-60°C according to the Primer Express 3.0 software (Life Technologies).

### Matrix degradation assay

Matrix degradation assay was performed as described earlier [58]. In brief, gelatin (Carl Roth, Karlsruhe, Germany) was fluorescently labeled with NHS-Rhodamine (Thermo Fisher Scientific, Waltham, MA). 15 mm coverslips were coated with labeled matrix solution, fixed in 0.5 % glutaraldehyde and washed with 70 % ethanol and RPMI Medium 1640 (Gibco BRL). Stable transfected, human MDA-MB-231-shLASP1 cells were seeded on coated coverslips with a density of 5×10^4^ cells/ml for 20 hours and fixed in 4 % (w/v) paraformaldehyde in PBS.

For immunofluorescence microscopy, cells were permeabilized with 0.1 % (w/v) Triton X-100 in PBS, and then stained with Oregon phalloidin green. Nuclei were visualized with DAPI (diluted 1:1000, Sigma-Aldrich, Taufkirchen, Germany).

Omnifocal analyses were done with a Biorevo BZ-9000 (Keyence, Neu-Isenburg, Germany). The obtained data were processed using Photoshop (Adobe Systems, San Jose, CA, USA). For quantification of matrix degradation, 100 randomly selected cells were analyzed for each condition by two independent persons. Cells with visible matrix degradation spots were counted as positive (most cells showed 15-20 degradation spots; see Figure 1B). Cells without any sign of matrix degradation were counted as negative.

### Preparation of serum-free conditioned medium

Cells were grown to approximately 80 % confluence in 75 cm^2^ plastic culture flasks containing 15 ml medium. The cultures were rinsed three times with Ca^2+^- and Mg^2+^- free PBS and then 5 ml of serum-free medium was added. After 24 h, the conditioned medium was collected, concentrated to 1 ml by SpeedVac (Eppendorf, Hamburg, Germany) and by Micron 10 kDa centrifugal filters (MerckMillipore, Darmstadt, Germany) and stored at −20°C. The remaining cells were counted with a cell counter (Scepter, MerckMillipore, Darmstadt, Germany), and 2 × 10^6^ cells were lysed in 100 μl RIPA buffer (25 mM Tris/HCl, 150 mM NaCl, 1 % NP40, 1 % Na-Deoxycholat, 0.1 % SDS) and stored at −20°C.

Protein concentration was determined by BCA protein assay (Pierce, Thermo Fisher Scientific). For Western Blot analysis, the conditioned medium was adapted to the corresponding cell count to guarantee equal loading.

### Zymography

Cell lysate of 5×10^4^ cells (20 μg protein) and conditioned medium of 1×10^6^ cells were subjected to electrophoresis under non-reducing conditions using either 10 % gelatin or 12 % casein containing gels (Thermo Fisher Scientific). After renaturation of the proteins by incubation of the gels in 100 ml Renaturing Buffer (Thermo Fisher Scientific) for 30 minutes twice, gels were incubated in 100 ml Developing Buffer (Thermo Fisher Scientific) at 37°C for 20 hours. The gels were stained with 0.1 % Coomassie Brilliant Blue in 30 % methanol/10 % acetic acid and de-stained in the same solution without Coomassie Brilliant Blue. Zones of proteolytic activity become visible as transparent bands in the blue gel. Human uterine fibroblasts (kind gift by Dr. U. Kämmerer; Department for Gynecology and Obstetrics, University Medical Clinic of Wuerzburg, Germany) were used as positive controls for MMP2, -3 and -9 activity.

### Affymetrix Chip and FunRich analysis

LASP1 knockdown and control cells were analyzed using GeneChip® PrimeView™ Human Gene Expression Arrays (Affymetrix). Algorithms for data analysis were written in R including different packages from the Bioconductor project (www.bioconductor.org). For the detection of differential expression the Limma package was used. Genes with a fold change > 1.5 were defined as differentially expressed. Functional clustering was performed using the software tool FunRich [31]. Raw data are deposited at http://www.ncbi.nlm.nih.gov/geo/query/acc.cgi?token=chctcmegdhmftox&acc=GSE77584.

### Analysis of public microarray data

Microarray data of primary breast cancer (n=1425) and metastases (n=29) were downloaded from Gene Expression Omnibus of NCBI (accession number: primary breast tumor GSE12276, GSE20685, GSE21653, GSE36771, GSE42568, GSE54002; breast cancer metastases GSE14017; all Affymetrix HG-U133plus2.0) and simultaneously normalized by Robust Multi-array Average [59] utilizing custom brainarray CDF file (v20.0.0 ENTREZG) representing for each gene an optimized probe-set on the microarray [60]. All data were controlled for correct annotation and plausibility manually. Samples were stratified by median LASP1 expression into a LASP1-high-expressing and LASP1-low-expressing group. Differential expression of the top 39 regulated genes in microarray analysis of MDA-MB-231-shLASP1 and all MMPs was assessed by calculation of log2 fold change between LASP1-low- and LASP1-high-expressing group of primary breast tumors and metastases, and between all breast tumors and metastases. Unpaired two-sided t-test to evaluate significance for differential expression and pearson squared were calculated on log2 transformed data.

### Luciferase reporter assay

Cignal dual-luciferase reporter assay for AP-1 transcriptional activity (Qiagen #336841) was performed following the manufacturer's instructions. Briefly, prior transfection, MDA-MB-231-shLASP1 cells were cultured for 3 days with and w/o doxycycline. Transfection was performed by Amaxa electroporation (Lonza, Cologne, Germany) with the mixture of an inducible AP-1 responsive luciferase construct and a constitutively expressing Renilla construct as internal standard. After transfection, 5x 10^4^ MDA-MB-231-shLASP1 cells per 24 well were seeded for another two days with and w/o doxycycline. Reporter assay was stopped by cell lysis in 100 μl passive lysis buffer (Promega #E1910, Mannheim, Germany). Firefly luciferase was measured by pipetting 20 μl of lysed cells to 100 μl LARII buffer (Promega) in 96-well opaque plates (Hartenstein, Wuerzburg, Germany) followed by adding 100 μl Stop&Glo reagent (Promega) and measuring Renilla luminescence using Wallac Victor^2^ (PerkinElmer, Waltham, MA). Firefly luciferase activity was normalized to Renilla luciferase activity.

### Pull-down of c-Jun

2×10^6^ MDA-MB-231-shLASP1 control cells were lysed in RIPA+-buffer (20 mM Tris pH 7.4, 150 mM NaCl, 1 % sodium-deoxycholate, 1 % Triton-X-100, 0.1 % SDS, 1 mM sodium orthovanadate, 10 mM sodium-pyrophosphate, 10 mM EDTA and protease inhibitor mixture). Immunoprecipitation was performed by rotation of 500 μl cell lysate with 2 μg His_6_-LASP1 for 2 h at 4°C followed by incubation with magnetic His-beads for 15 min at 4°C. For GST-pulldown, 500 μl cell lysate was incubated with 2 μg GST-tagged LASP1 for 1 h at 4°C followed by incubation with 50 μl GST-sepharose beads (GE Healthcare) for 60 min. The immune complexes were washed two times with ice-cold PBS buffer and prepared for SDS-PAGE.

### Preparation of nuclear (and cytosolic) cell fraction

MDA-MB-231-shLASP1 cells were harvested at 80 % confluence through trypsination. Isolation of nuclei and cytosol was carried out using NE-PER Nuclear and Cytoplasmic Extraction Reagents (Pierce, Bonn, Germany) following the manufacturer's instructions.

### Statistics

Unpaired two-tailed Student's t test for independent groups was applied to determine effects of siRNA knockdown on MMP expression and secretion as well as on reporter assay data. Results were considered significant at p<0.05 (*p<0.05; **p<0.01; ***p<0.001; n.s. not significant).

## SUPPLEMENTARY FIGURE AND TABLES






