# Supplementary material for: Regulation of matrix metalloproteinases (MMPs) expression and secretion in MDA-MB-231 breast cancer cells by LIM and SH3 protein 1 (LASP1)
Source: Oncotarget. 2016 Aug 31;7(39):64244–59. doi: 10.18632/oncotarget.11720 (PMC5325439; doi:10.18632/oncotarget.11720)
Supplement: Supplementary file 1 [file oncotarget-07-64244-s001.pdf]

Regulation of matrix metalloproteinases (MMPs) expression and secretion in MDA-MB-231 breast cancer cells by LIM and SH3 protein 1 (LASP1)

SUPPLEMENTARY FIGURE AND TABLES

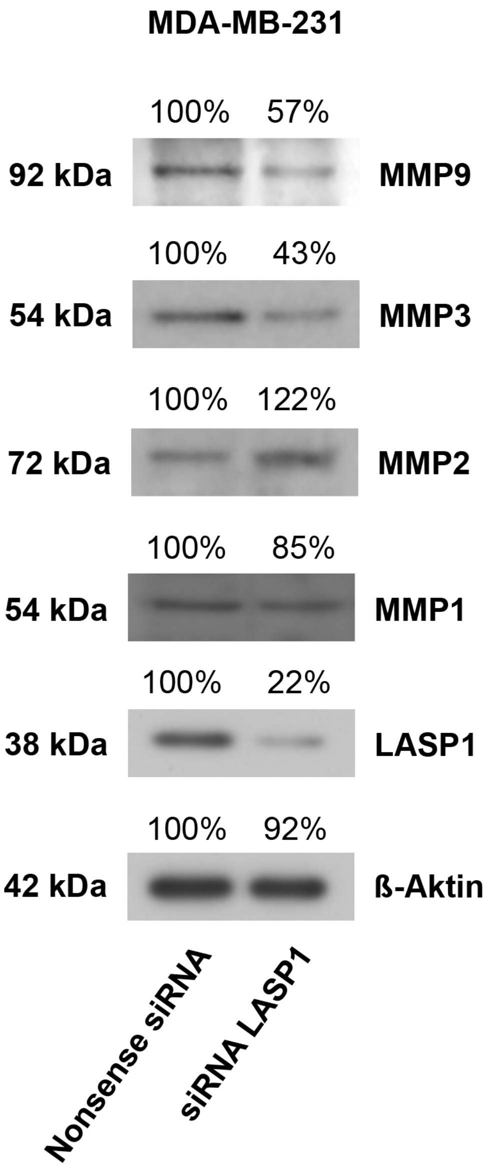

Supplementary Figure S1: Western Blot analysis of LASP1, β-actin (loading control) and MMPs of MDA-MB-231 cells transfected with nonsense siRNA or LASP1 specific siRNA according to *Materials and Methods*. Western Blot analysis confirmed LASP1 knockdown concomitant with impaired MMP1, -3, and -9 protein expression. Signals were quantified using ImageJ densitometric scanning software and relative protein amount was calculated by normalization with β-actin. Numbers above the blots indicate percentage of protein expression after LASP1 knockdown (siRNA LASP1) compared to 100% control (nonsense siRNA). Data are representative for three independent experiments.

Supplementary Table S1: List of 39 key genes regulated by LASP1 log2FC = LASP1-depleted/LASP1-control.

Supplementary File 1

Supplementary Table S2: Analysis of public microarray data from primary breast tumors. Differential expression of 38 of the top 39 LASP1 regulated genes assessed in MDA-MB-231-shLASP1 microarray analysis was evaluated comparing samples with low and high LASP1 expression stratified by median expression. The 39th gene was not mapped in the used CDF file (*FAM23A*). Results were compared to the MDA-MB-231-shLASP1 microarray analysis.

Supplementary File 2

Supplementary Table S3: Primer sequences used for qRT-PCR

Gene name	fw-primer	rev-primer
hLASP1	AAAACCTTCGCCTCAAGCAA	AGCAGGTCAGCAAAGAATTTATAGC
hMMP1	GCTAACCTTTGATGCTATAATACGATTC	GGATTTGTGCGCATGTAGAATC
hMMP2	CCCCCAAAACGGACAAAGA	CCTTCAGCACAAACAGGTTGC
hMMP3	CAACA AGAGCTAAGTAAAGCCAGTGG	CTAGATATTTCTGAACAAGGTTCATGCT
hMMP9	TCCCTGGAGACCTGAGAACCA	CGACTCTC ACGCATCTCTG
hMMP14	GTCAGGAATGAGGATCTGAATGG	CGAGGGGTCACTGGAATGC
hRPL0	CATCTACAACCCTGAAGTGCTTGAT	CAATCTGCAGACAGACACTGGC
