# Supplementary material for: Regulation of matrix metalloproteinases (MMPs) expression and secretion in MDA-MB-231 breast cancer cells by LIM and SH3 protein 1 (LASP1)
Source: Oncotarget. 2016 Aug 31;7(39):64244–59. doi: 10.18632/oncotarget.11720 (PMC5325439; doi:10.18632/oncotarget.11720)
Supplement: Supplementary file 2 [file oncotarget-07-64244-s002.docx]

**Supplementary Table 1:** List of 39 key genes regulated by LASP1

log2FC = LASP1-depleted/LASP1-control.

| **Gene Symbol** | **Ensembl** | **log2FC** |  |
| --- | --- | --- | --- |
|  |  |  |  |
|  |  |  |  |
| LASP1 | ENSG00000002834 | -0,777735444 |  |
| LASP1 | ENSG00000002834 | -2,081569139 |  |
| LASP1 | ENSG00000002834 | -2,434279723 |  |
| ZNF582 | ENSG00000018869 | -0,612862807 |  |
| TFB1M | ENSG00000029639 | -1,237925697 |  |
| VCAN | ENSG00000038427 | -0,592507196 |  |
| SPAG4 | ENSG00000061656 | 0,589629633 |  |
| BCL3 | ENSG00000069399 | 0,757668418 |  |
| SEMA3C | ENSG00000075223 | -0,613522536 |  |
| ABCC6 | ENSG00000091262 | 0,620394911 |  |
| CDK6 | ENSG00000105810 | -0,645503119 |  |
| CDK6 | ENSG00000105810 | -0,716656158 |  |
| ALDOC | ENSG00000109107 | 0,694364127 |  |
| FAM46A | ENSG00000112773 | 0,590862935 |  |
| SCP2 | ENSG00000116171 | 0,631381342 |  |
| KLF9 | ENSG00000119138 | 0,680147649 |  |
| PDZD2 | ENSG00000133401 | -0,612159518 |  |
| PDZD2 | ENSG00000133401 | -0,612911650 |  |
| SLC2A12 | ENSG00000146411 | 0,702819704 |  |
| PTGES | ENSG00000148344 | 0,602924691 |  |
| TMEM236 | ENSG00000148483 | -0,619670829 |  |
| EIF4EBP2 | ENSG00000148730 | -0,606261264 |  |
| MMP3 | ENSG00000149968 | -0,823940305 |  |
| MBNL1 | ENSG00000152601 | -0,593756268 |  |
| MBNL1 | ENSG00000152601 | -0,658923209 |  |
| GPR180 | ENSG00000152749 | -1,081233583 |  |
| NR4A2 | ENSG00000153234 | 0,722722116 |  |
| EYA3 | ENSG00000158161 | -0,613047976 |  |
| GBP2 | ENSG00000162645 | 0,677615997 |  |
| SLC22A15 | ENSG00000163393 | -0,628987599 |  |
| MFSD8 | ENSG00000164073 | -0,678619764 |  |
| FOS | ENSG00000170345 | 0,691198977 |  |
| FOS | ENSG00000170345 | 0,679105153 |  |
| PAQR8 | ENSG00000170915 | 0,633291595 |  |
| PDE7B | ENSG00000171408 | 0,757191029 |  |
| MCC | ENSG00000171444 | -0,612554166 |  |
| IFITM2 | ENSG00000185201 | 0,733322784 |  |
| METTL7A | ENSG00000185432 | 0,762266044 |  |
| PBX1 | ENSG00000185630 | 0,783815654 |  |
| IFITM1 | ENSG00000185885 | 0,698062939 |  |
| C11orf96 | ENSG00000187479 | 0,637544191 |  |
| PEAR1 | ENSG00000187800 | -0,603299715 |  |
| BLOC1S2 | ENSG00000196072 | -0,825715588 |  |
| BLOC1S2 | ENSG00000196072 | -0,943640745 |  |
| MMP1 | ENSG00000196611 | -0,663132175 |  |
| MMP1 | ENSG00000196611 | -0,750457082 |  |
| ZNF138 | ENSG00000197008 | -0,643613617 |  |
|  |  |  |  |
|  |  |  |  |
