# Supplementary material for: Regulation of matrix metalloproteinases (MMPs) expression and secretion in MDA-MB-231 breast cancer cells by LIM and SH3 protein 1 (LASP1)
Source: Oncotarget. 2016 Aug 31;7(39):64244–59. doi: 10.18632/oncotarget.11720 (PMC5325439; doi:10.18632/oncotarget.11720)
Supplement: Supplementary file 3 [file oncotarget-07-64244-s003.docx]

**Supplementary Table 2: Analysis of public microarray data from primary breast tumors.** Differential expression of 38 of the top 39 LASP1 regulated genes assessed in MDA-MB-231-shLASP1 microarray analysis was evaluated comparing samples with low and high LASP1 expression stratified by median expression. The 39th gene was not mapped in the used CDF file (*FAM23A*). Results were compared to the MDA-MB-231-shLASP1 microarray analysis

| **Gene symbol** | **Entrez gene ID** | **log2FC**  (low vs. high LASP1 expressing primary breast tumors) | **Pearson's r** | **p-Value** | **Significant and consistent with MDA-MB-231-shLASP1 results?** | **log2FC** (knockdown vs. control MDA-MB-231-shLASP1) |
| --- | --- | --- | --- | --- | --- | --- |
|  |  |  |  |  |  |  |
| AG2 | 387763_at | -0.50627839 | 0.10668176 | 0.00014647 | NO | 0.63754419 |
| SLC22A15 | 55356_at | -0.27862755 | 0.08715332 | 0.01062022 | YES | -0.6289876 |
| VCAN | 1462_at | -1.01379257 | 0.18464884 | 1.6787E-10 | YES | -0.5925072 |
| GPR180 | 160897_at | 0.51315746 | -0.21529258 | 8.013E-09 | NO | -1.08123358 |
| GBP2 | 2634_at | -0.44088284 | 0.07280967 | 0.00037806 | NO | 0.677616 |
| FOS | 2353_at | -0.86708435 | 0.15121758 | 7.1726E-07 | NO | 0.69119898 |
| EIF4EBP2 | 1979_at | 0.14577734 | -0.06351266 | 0.02318651 | NO | -0.60626126 |
| EYA3 | 2140_at | 0.18681601 | -0.20960994 | 2.0275E-11 | NO | -0.61304798 |
| MBNL1 | 4154_at | 0.01286971 | 0.00613648 | 0.63239278 | NO | -0.65892321 |
| ABCC6 | 368_at | -0.19308825 | 0.06621641 | 0.01441417 | NO | 0.62039491 |
| PTGES | 9536_at | -0.30243142 | 0.03448536 | 0.05768878 | NO | 0.60292469 |
| SLC2A12 | 154091_at | 0.30495808 | -0.12384886 | 0.0053196 | YES | 0.7028197 |
| LASP1 | 3927_at | -2.62667534 | 1 | 4.009E-130 | YES | -2.43427972 |
| ZNF582 | 147948_at | 0.01611815 | -0.02972688 | 0.62981018 | NO | -0.61286281 |
| BLOC1S2 | 282991_at | 0.00745498 | -0.03167215 | 0.47164937 | NO | -0.94364075 |
| METTL7A | 25840_at | -0.28116165 | 0.05941746 | 0.00311658 | NO | 0.76226604 |
| PDE7B | 27115_at | -0.00871829 | 0.03499402 | 0.32564697 | NO | 0.75719103 |
| ALDOC | 230_at | -0.0317543 | 0.0118615 | 0.90948359 | NO | 0.69436413 |
| IFITM1 | 8519_at | -0.28658794 | 0.05862769 | 0.00057639 | NO | 0.69806294 |
| PBX1 | 5087_at | -0.65080852 | 0.24813766 | 1.4552E-13 | NO | 0.78381565 |
| IFITM2 | 10581_at | -0.78000738 | 0.22304378 | 4.439E-14 | NO | 0.73332278 |
| ZNF138 | 7697_at | -0.0276125 | -0.00898966 | 0.95852313 | NO | -0.64361362 |
| CDK6 | 1021_at | 0.89665375 | -0.21511936 | 4.0264E-11 | NO | -0.71665616 |
| BCL3 | 602_at | -0.3009053 | 0.20544812 | 2.3215E-07 | NO | 0.75766842 |
| MMP3 | 4314_at | -0.20630164 | -0.01958613 | 0.46515273 | NO | -0.82394031 |
| PDZD2 | 23037_at | -0.28269789 | 0.07192516 | 0.01760026 | YES | -0.61291165 |
| NR4A2 | 4929_at | -0.32180522 | 0.07085914 | 0.00435486 | NO | 0.72272212 |
| PAQR8 | 85315_at | 0.40961154 | -0.15477329 | 3.8127E-05 | YES | 0.6332916 |
| KLF9 | 687_at | -0.29727088 | 0.17768857 | 2.7267E-06 | NO | 0.68014765 |
| FAM46A | 55603_at | 0.44721284 | -0.07795567 | 0.03082139 | YES | 0.59086294 |
| MFSD8 | 256471_at | -0.07408536 | 0.03148408 | 0.07923345 | NO | -0.67861976 |
| MCC | 4163_at | 0.15870137 | -0.02713154 | 0.37282933 | NO | -0.61255417 |
| TFB1M | 51106_at | 0.32656555 | -0.2064525 | 3.1715E-08 | NO | -1.2379257 |
| SCP2 | 6342_at | -0.18368244 | 0.09855108 | 0.00080362 | NO | 0.63138134 |
| SEMA3C | 10512_at | -0.43949554 | 0.17090699 | 2.1692E-08 | YES | -0.61352254 |
| MMP1 | 4312_at | 0.06662966 | -0.04619948 | 0.25422239 | NO | -0.75045708 |
| PEAR1 | 375033_at | -0.38607376 | 0.13619177 | 2.8796E-08 | YES | -0.60329972 |
| SPAG4 | 6676_at | -0.27791124 | 0.03402438 | 0.06855897 | NO | 0.58962963 |
|  |  |  |  |  |  |  |
| MMP1 | 4312_at | 0.06662966 | -0.04619948 | 0.25422239 | NO | -0.75045708 |
| MMP2 | 4313_at | -1.11157124 | 0.17332119 | 3.0252E-10 | NO | 0.21412481 |
| MMP3 | 4314_at | -0.20630164 | -0.01958613 | 0.46515273 | NO | -0.82394031 |
| MMP7 | 4316_at | 2.53038635 | -0.2188465 | 3.9113E-09 | NO | -0.04264434 |
| MMP8 | 4317_at | -0.00759422 | -0.02059978 | 0.49768069 |  |  |
| MMP9 | 4318_at | -0.48082019 | 0.07979713 | 0.00315809 | YES | -0.92599942 |
| MMP10 | 4319_at | -0.98229265 | 0.07615481 | 0.00690572 |  |  |
| MMP11 | 4320_at | -1.13844255 | 0.20686192 | 5.5809E-12 |  |  |
| MMP12 | 4321_at | 0.92136066 | -0.11325334 | 0.00062589 | YES | 0.18903382 |
| MMP13 | 4322_at | -0.60521653 | 0.05610252 | 0.00222232 | YES | -0.04264434 |
| MMP14 | 4323_at | -0.54371553 | 0.14907703 | 8.7441E-05 | NO | 0.01435529 |
| MMP15 | 4324_at | -0.16522079 | 0.1538492 | 0.00069686 |  |  |
| MMP16 | 4325_at | 0.13500311 | -0.04348542 | 0.0753754 |  |  |
| MMP17 | 4326_at | -0.13161633 | 0.08464748 | 0.01625228 |  |  |
| MMP19 | 4327_at | -0.17343927 | 0.10124433 | 0.00038895 | NO | 0.02856915 |
| MMP20 | 9313_at | 0.47590278 | -0.17740964 | 1.3061E-05 |  |  |
| MMP21 | 118856_at | 0.01133785 | 0.0320724 | 0.59284919 |  |  |
| MMP24 | 10893_at | 0.10173638 | -0.04898146 | 0.03324044 |  |  |
| MMP25 | 64386_at | 0.06079022 | -0.03504621 | 0.18175636 |  |  |
| MMP26 | 56547_at | 0.10901096 | -0.01378281 | 0.06110649 |  |  |
| MMP27 | 64066_at | 0.16092047 | -0.09231631 | 0.00638094 |  |  |
| MMP28 | 79148_at | -0.04195208 | 0.09020846 | 0.06826404 |  |  |
